# Nonlinear light conversion and infrared photodetection with laser-printed plasmonic metasurfaces supporting bound states in the continuum

**DOI:** 10.1038/s41377-025-02040-4

**Published:** 2026-01-02

**Authors:** Dmitrii V. Pavlov, Kseniia A. Sergeeva, Albert A. Seredin, Artem B. Cherepakhin, Aleksandr A. Sergeev, Anastasiia V. Sokolova, Yuri N. Kulchin, Alexey Yu. Zhizhchenko, Mihail I. Petrov, Aleksandr A. Kuchmizhak, Andrey L. Rogach

**Affiliations:** 1 Institute of Automation and Control Processes, Far Eastern Branch, Russian Academy of Sciences, 5 Radio Str., Vladivostok, 690041 Russia; 2 Department of Materials Science and Engineering, and Centre for Functional Photonics (CFP), City University of Hong Kong, Kowloon, Hong Kong SAR 999077 China; 3 School of Physics and Engineering, ITMO University, Saint-Petersburg, 197101 Russia; 4 Department of Physics, Hong Kong University of Science and Technology, Clear Water Bay Rd, Kowloon, Hong Kong SAR 999077 China; 5 Pacific Quantum Center, Far Eastern Federal University, 8 Sukhanova str., Vladivostok, 690041 Russia

**Keywords:** Mid-infrared photonics, High-harmonic generation, Photonic devices, Optoelectronic devices and components

## Abstract

Plasmonic metasurfaces supporting high-quality (Q) resonances offer unprecedented ways for controlling light-matter interaction at the nanoscale, yet scalable fabrication of such sophisticated nanostructures still relies on expensive and multi-step fabrication routes, hindering their practical application. Here, we produced plasmonic metasurfaces composed of the regular arrangement of hollow protruding nanobumps via direct femtosecond laser patterning of thin gold films. By using comprehensive optical modeling, infrared spectroscopy and angle-resolved third harmonic generation experiments, we justified that such laser-printed nanostructures support symmetry-protected plasmonic quasi-bound states in the continuum (qBIC) with a measured Q-factor up to 20. Moreover, under critical coupling conditions that match the radiative and nonradiative losses of the high-Q mode, the metasurfaces demonstrate the third harmonic generation enhanced by a factor of ≈10^5^ as compared to the smooth Au film benchmark, proving structure efficiency for nonlinear conversion. Finally, by taking advantage of the simplicity and straightforward character of the laser printing process, we realized a field-effect transistor device with HgTe quantum dots as an active medium and qBIC-supporting plasmonic metasurface imprinted over drain and source electrodes. The resulting metasurface-empowered device operates at 200 K and 5 V bias voltage and demonstrates superior specific detectivity around 8.7 × 10^11^ at the plasmonic-qBIC spectral region and fast response time, holding promise for the realization of advanced shortwave infrared photodetectors.

## Abstract (web summary)

Laser-printed gold metasurface supporting symmetry-protected plasmonic quasi-bound states in the continuum (qBIC) was fabricated, comprehensively characterized and applied to demonstrate the first qBIC-empowered field-effect transistor device with HgTe quantum dot active medium.

## Graphical abstract

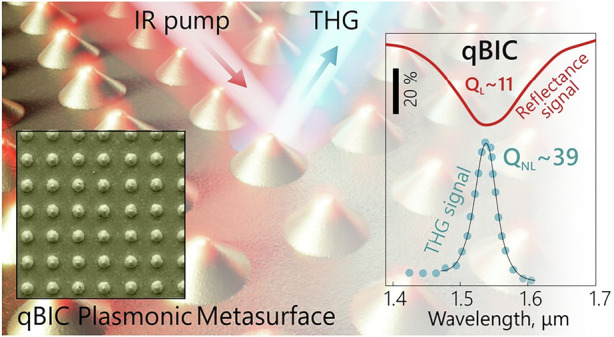

## Introduction

Excitation of resonant oscillations of free-electron plasma at the metal-dielectric interface induced by an external electromagnetic field, namely surface plasmon-polariton (SPP) waves, has revolutionized many research fields, including optoelectronics, nanophotonics, nonlinear optics, and sensors, offering deep subwavelength light confinement and unique pathways for resonant light-matter interaction at the nanoscale^1–3^. Such oscillations can be induced in the form of propagating interface waves or excited in isolated nanostructures that are typically made of noble metals and support geometry-dependent localized surface plasmon resonances (LSPRs)^4^. At the same time, despite the good combination of optical properties and chemical stability, the inherently large Ohmic losses of common plasmon-active metals lead to low quality (Q) factors of the LSPR modes, limiting the practical applicability and efficiency of the related nanostructures. Coherent matching of electromagnetic waves scattered by isolated plasmonic nanoresonators with propagating/localized SPPs can be realized in specially designed regular nanostructure arrangements, also referred to as plasmonic metasurfaces^5–7^. These metasurfaces allow one to partially compensate for overall losses of the resonant system by coupling with diffraction channels or by introducing interference suppression effects, resulting in the excitation of collective plasmon modes with high finesse^8,9^. Meanwhile, excitation of the lattice plasmon modes in the substrate-supported plasmonic nanostructure arrays typically requires inconvenient angular excitation or refractive index matching conditions to be realized, complicating their practical usage^8^.

Bound states in the continuum (BIC) represent a specific class of high-Q modes that have been intensively studied over the last decade in both fundamental and applied aspects^10–12^. Excitation of these low-loss modes ensures spectrally narrow optical response combined with a strong near-field light localization, holding promise for optical sensors, resonant filters, nonlinear conversion and active photonic devices. Metasurfaces supporting BICs mostly rely on common semiconductor materials (Si, Ge, TiO_2_, etc.), where strong light confinement inside low-loss host nanostructures allows achieving high efficiency in nonlinear conversion^13^, while high-Q resonances are found useful for optical detection^14,15^. Recently, intensive research into plasmonic systems supporting BIC has started^16–18^, demonstrating particular applications in molecular detection^19^, perfect absorption^20^, and photoluminescence enhancement^21,22^. Indeed, dielectric structures offer higher Q-factors due to their low intrinsic losses compared to plasmonic structures. This is primarily because dielectrics do not suffer from ohmic losses, which are a significant source of energy dissipation in metals used for plasmonic systems. However, plasmonic structures have a much smaller mode volume due to the strong localization of electromagnetic fields near the metal-dielectric interface, which enables them to confine light to subwavelength scales. This trade-off between high Q-factors in dielectric systems and low mode volumes in plasmonic systems defines their respective advantages and applications in optical technologies. A more detailed description of possible theoretical values of the Q-factor in plasmonic and dielectric metasurfaces can be found in refs. ^16,23,24^.

Achieving BIC mode excitation with plasmonic metasurfaces typically requires resonant light-matter interaction realized through sophisticated three-dimensional (3D) designs of the isolated plasmonic meta-atoms ordered in the lattices. Complicated designs are commonly produced with costly multi-step lithography-based techniques, primarily used for planar nanostructure fabrication, while emerging methods such as template-assisted charge aerosol printing^25,26^ or multiphoton laser polymerization followed by metal deposition^17,19^ cannot be considered less expensive and more mechanically/thermally robust and scalable solutions pushing the quest for better, practically relevant fabrication routes.

**Fig. 1:**
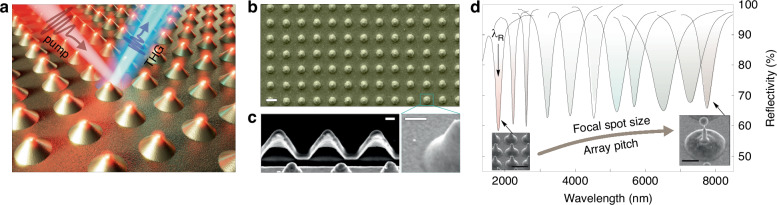
BIC-supporting plasmonic metasurface. **a** Sketch showing THG process under near-IR pump of the plasmonic metasurface composed of ordered array of laser-printed gold nanobumps. **b** Representative top-view SEM image of the gold nanobump array produced at an array pitch of Λ = 1.5 µm and applied pulse energy of *E* = 2.7 nJ, as well as **c** cross-sectional FIB cut showing hollow structure of the nanobumps. Scale bars on (**b**) and (**c**) correspond to 1 and 0.2 µm, respectively. **d** Wide-range FTIR spectra of the plasmonic nanobump arrays showing near-to-mid IR tunability of the collective plasmonic mode spectral position by varying the array pitch Λ and the nanobump size, as illustrated in the insets

In this paper, we propose an efficient single-step approach for the fabrication of BIC-supporting plasmonic metasurfaces through direct femtosecond (fs) laser patterning on a thin glass-supporting gold film in an ablation-free regime. The metasurface was composed of the regular array of hollow protruding nanobumps supporting the BIC mode at infrared (IR) wavelengths and providing facile tunability of the resonant wavelength through the variation of the nanostructure geometry and spacing. By combining Fourier transform infrared (FTIR) spectroscopy and angle-resolved third-harmonic generation (THG) experiments (Fig. 1a) with comprehensive optical modeling, we shed light on the origin of the observed resonant optical and nonlinear optical responses of the produced metasurfaces supporting symmetry-protected a plasmonic BIC mode with a measured Q-factor of up to 40. Under optimal critical coupling conditions providing matching of the radiative and non-radiative mode losses, the metasurfaces demonstrated the THG signal yield enhanced by a factor of ≈10^5^ as compared to the smooth Au film benchmark, proving BIC-empowered light localization boosting efficient nonlinear conversion. Moreover, we realized a first BIC-empowered design of field-effect transistor (FET) device based on the HgTe quantum dot (QD) layer capped over the plasmonic metasurface, which was directly imprinted over drain and source electrodes. The resulting metasurface-empowered device operating at 200 K and 5 V bias voltage demonstrated superior specific detectivity around 8.7 × 10^11^ at the plasmonic-BIC spectral region, holding promise for the realization of advanced shortwave infrared (SWIR) photodetectors.

## Results and discussion

### Plasmonic BICs in laser-printed nanobump arrays

Rectangular arrays of nanobumps (200 × 200 µm^2^ in size) were first laser imprinted on the surface of a 50-nm-thick glass-supported Au film following the common ablation-free procedure described elsewhere^22,27^. Briefly, the local section of the laser-irradiated Au film undergoes melting and thermomechanical relaxation from the supporting substrate, leading to the formation of hollow nanobumps upon resolidification^28^. It is worth mentioning that this ablation-free procedure produces protruding Au nanostructures with high morphological smoothness (compared to the pristine sputtered film; Fig. 1b), which is potentially beneficial for minimizing the scattering losses. A representative scanning electron microscopy (SEM) image of the fabricated array confirms this statement (Fig. 1c), while the hollow structure of the nanobumps is unveiled by the corresponding image of the cross-section cut by focused ion beam milling (FIB; Fig. 1c). Ordered rectangular array of nanobumps was previously shown to demonstrate a collective plasmonic response in the IR part of the spectrum that manifests itself through the appearance of the reflection dip (see Fig. 1d) with a position *λ*_*R*_ depending on both the array pitch Λ and the energy-governed nanobump geometry^22^. Starting from the typical pitch of Λ = 1.2 µm and systematically increasing the size of the nanobumps through the size of the laser focal spot (controlled by the numerical aperture (NA) of the focusing lens), and enlarging the distance between them (up to Λ = 4 µm), one can gradually redshift the resonance position *λ*_*R*_ over a wide spectral range spanning from 1.3 to 8 µm as demonstrated by a series of FTIR reflectance spectra in Fig. 1d. As can be seen, the reflectance dip shows an average amplitude of about 40% and an average Q-factor Q_L_ = Δ*λ*_*R*_/*λ*_*R*_ ranging from 12 to 17. Moreover, an ablation-free fabrication procedure allows to achieve high reproducibility of the nanobump geometry, ensuring a small variation of the *λ*_*R*_ under cyclic fabrication (see Fig. S1 in Supplementary Materials).

**Fig. 2:**
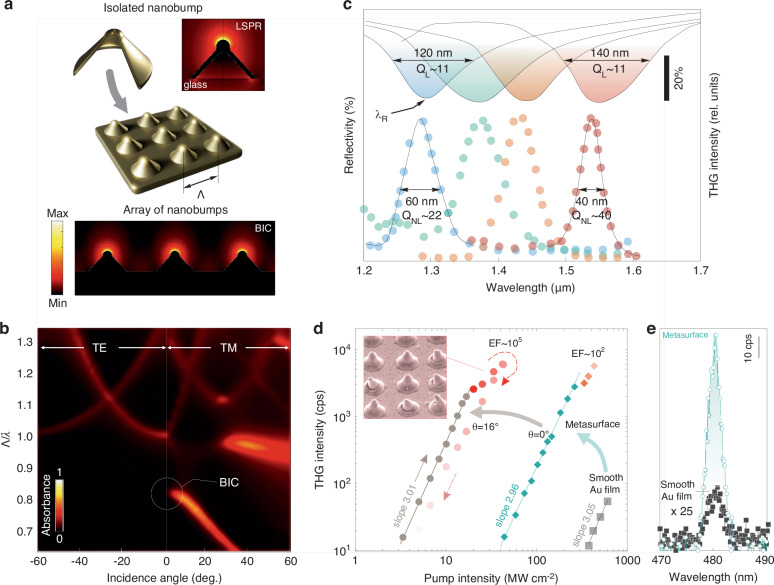
Optical spectroscopy and THG from BIC-supporting plasmonic metasurfaces. **a** Sketch of an isolated nanobump and its ordered arrays, as well as calculated intensity distribution upon excitation of the LSPR in the isolated structure (at *λ*_*R*_ = 2.54 µm) and BIC mode in the array of such structures (at *λ*_*R*_ = 1.47 µm). The following parameters of the nanobump were used for simulations: height of 0.42 µm, the base radius of 0.4 µm and pitch size of Λ = 1.2 µm. **b** Absorption map calculated for the aforementioned nanobump array under TE- and TM-polarized excitation for different incidence angles *θ*. Vertical axis is plotted as the normalized frequency Λ/*λ*. **c** FTIR spectra of several produced nanobump arrays (top solid curves), as well as corresponding dependencies of THG yield I_3ω_ measured for each array as a function of fundamental harmonic wavelength *λ*_*ω*_ (bottom dotted curves). **d** Comparative power dependencies of the THG signal obtained from the smooth Au film and from plasmonic metasurface under its resonant excitation (*λ*_*ω*_ = *λ*_*R*_) and at *θ* = 0° and 16°. **e** Spectra of the THG signal measured from the smooth Au film and plasmonic metasurface under its resonant excitation at *θ* = 0°

The physical origin of the reflectance dip is associated with a quasi-BIC excited in the plasmonic nanostructure array. In order to shed light on the features of this mode, we carried out comprehensive optical modeling considering the realistic 3D geometry of the representative isolated nanobump (with a height of 0.42 µm and a base radius of 0.4 µm) sketched in Fig. 2a. Note that such an isolated nanostructure can support its own resonant optical modes associated with geometry-dependent LSPRs. For the mentioned geometry of the nanobump placed on the glass substrate, our simulations give the lossy localized mode with a dipole moment oriented normally to the interface with a Q-factor around Q ≈ 3 at *λ*_*R*_ = 2.54 µm. The electromagnetic field is predominantly localized at the top of the structure, as shown in Fig. 2a (right panel). The situation changes dramatically when such nanobumps are connected with smooth sections of the metal film and arranged into a two-dimensional array with a period Λ. The interaction between the isolated nanostructures through the SPP waves leads to the formation of a collective mode. The typical absorption map calculated for the mentioned nanobumps in the rectangular array with Λ = 1.2 µm as a function of the incidence angle *θ* for TM- or TE-polarized plane waves and normalized frequency Λ/*λ* is shown in Fig. 2b, where, under the TM-polarized excitation, one can notice a pronounced low-frequency fundamental mode at *λ*_*R*_ with strong absorption. This mode is of a BIC-type inherent to the structure of in-phase vertical electric dipoles, as shown in Fig. 2a. The collective interaction between them leads to a strong blue shift of the fundamental mode from *λ*_*R*_ = 2.54 µm (for an isolated nanobump) to 1.47 µm (Λ/*λ* = 0.82 at Λ = 1.2 µm; Fig. 2b) at a small angle near the Γ-point. Moreover, its BIC origin is reflected in the nearly zero absorption exactly at the Γ-point, a distinctive feature of the symmetry-protected BIC modes with vanishing radiative losses. The performed simulations also proved that the field distribution of BIC mode has a typical antisymmetric character^29^ (Fig. S2). Finally, simulations also clearly demonstrated the progressive redshift of the BIC mode position once the size of the nanobumps in the array or its periodicity Λ increases, as well as confirmed the nature of the collective interaction between the nanobumps in the array through the SPP waves rather than the waves scattered to the free space (Fig. S3). Finally, for both excitation polarizations, one can also identify several resonant modes at Λ/*λ* > 1, which are attributed to the lattice plasmonic modes originating from the coupling between the LSPRs and diffraction channels^8^.

### THG empowered by the plasmonic BICs of the laser-printed metasurface

According to the performed simulations (Fig. 2a), excitation of the plasmonic BIC should result in enhanced absorption of the incident light accompanied by the strong electromagnetic field localization at the air-gold interface. In its turn, enhanced near fields can boost nonlinear optical effects such as higher harmonic generation^30^, which can be used for detailed characterization of the observed BIC mode. In this study, we specifically focus on the THG as a coherent three-photon process originating from the nonlinear polarizability ${P}_{i}^{(3\omega )}={\varepsilon }_{0}{\chi }_{{ijkl}}^{(3)}{\sum }_{{jkl}}{E}_{j}^{(\omega )}{E}_{k}^{(\omega )}{E}_{l}^{(\omega )}$, where ${\chi }^{(3)}$ is the third-order nonlinear susceptibility of gold ${\chi }^{(3)}=2.45\,\cdot \,{10}^{-19}{V}^{2}{m}^{-2}$ ^31^, and ${E}_{i=x,y,z}^{(\omega )}$ are the local field components at the fundamental wavelength. The nanobump fabrication parameters were accurately tailored (1.0 < Λ < 1.3 µm; 2.5 < pulse energy < 2.9 nJ; NA = 0.42) to achieve the plasmonic response within 1.2–1.55 µm, matching the available tuning range of our optical parametric amplifier (OPA) system (see “Materials and methods”). The BIC wavelength was then optically probed by detecting the THG yield I_3*ω*_ as function of the fundamental harmonic wavelength *λ*_*ω*_ scanning across the FTIR-identified mode wavelength *λ*_*R*_.

The experiments were first carried out under normally incident (*θ* = 0°) low-intense (P_*ω*_ = 45 mW) pump, while their results measured for several arrays are shown in Fig. 2c as markers. As can be seen, in each case the I_3*ω*_(*λ*_*ω*_) dependence demonstrates a resonant-like behavior with the maximal THG yield observed at *λ*_*ω*_ = *λ*_*R*_ (see Fig. 2c). Some spectral mismatch (±30 nm) between the maximal THG yield and the reflectance dip can be explained by local variations of the shape-dependent resonant properties of the isolated nanobumps within the array, as well as the more local nature of the THG signal pump with its focal plane beam size around 50 µm as compared to the FTIR measurements collecting low-noise signal from 200 × 200 µm^2^ surface area. At the same time, nonlinear signal systematically demonstrates at least twice narrower resonance linewidth of the I_3*ω*_(*λ*_*ω*_) dependence, yielding a larger Q-factor up to 40. Potential explanation of such a phenomenon is the wide range of incident angles appearing upon probing the plasmonic metasurface with a focused light^18,32^, which is the case for the performed FTIR measurements carried out with a reflective-type objective having an NA of 0.5. Nonlinear THG experiments ensure excitation of the metasurface with a more collimated light beam, leading to larger detected resonance Q-factors. The average THG yield of the plasmonic metasurface under resonant excitation and the normally incident pump (*θ* = 0°) versus the pump intensity demonstrates clear three-photon behavior with the slope ≈2.96 preserved up to 120 mW pump power (green curves in Fig. 2d). At the higher intensities, the dependence deviates from cubic law due to optically increased thermal effects which may be followed by consequent deformation of bumps as can be identified in the SEM image (Fig. 2d, inset). The laser-printed plasmonic metasurface provides nearly two orders of magnitude enhancement factor (EF) of the THG process as compared to the non-patterned smooth Au film, with no evident modifications of the THG spectrum (Fig. 2e).

**Fig. 3:**
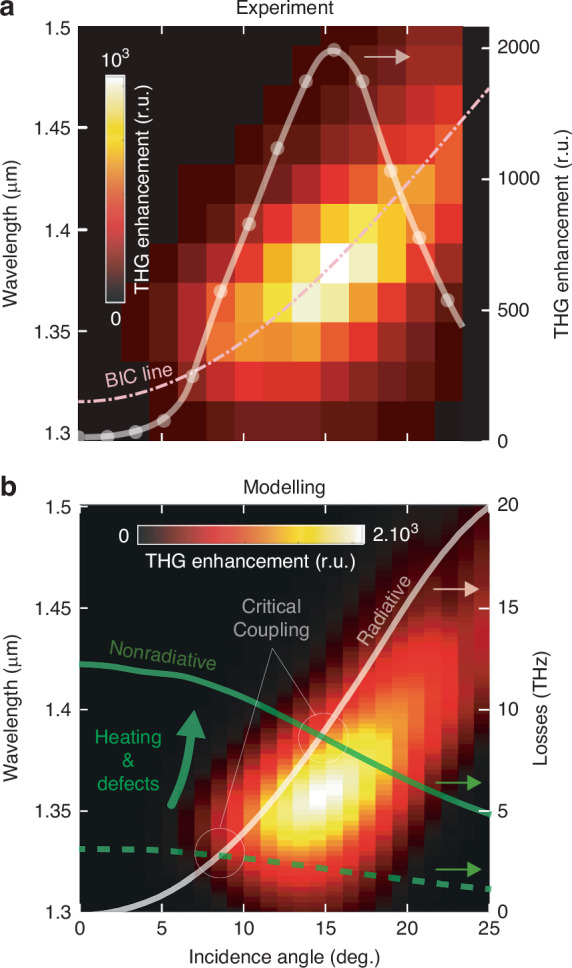
Critical coupling condition for enhancing THG. Measured (**a**) and calculated (**b**) dependencies of THG yield of the representative nanobump array (*λ*_*R*_ = 1.32 µm; Λ *=* 1 µm) on the pump laser wavelength and incidence angle *θ* under TM-polarized excitation. Markers on (**b**) trace the variation of the THG signal intensity versus incidence angle *θ* under resonant excitation *λ*_*ω*_ = *λ*_*R*_. Curves on (**b**) provide calculated radiative (white line) and non-radiative (green lines) decay rates of the plasmonic BIC mode versus *θ*. Non-radiative losses were calculated considering only the Ohmic losses derived from the optical constants of gold (green dashed curve), as well as additional losses due to fabrication defects of the array and thermally increased Ohmic losses (green solid curve)

Analysis of the absorption coefficient of the plasmonic BIC mode plotted in Fig. 2b identifies the maximal values achieved at a certain angle *θ*_cr_, which corresponds to the critical coupling condition^33,34^, when the radiative and non-radiative counterparts of the mode losses become equal. At the critical angle, the electromagnetic energy accumulated in the plasmonic BIC reaches its maximal value, leading to enhancement of the local electromagnetic field amplitude and boosting the nonlinear response. In this respect, we further systematically studied the THG yield of the nanobump array with the BIC at *λ*_*R*_ = 1.32 µm as a function of the pump wavelength and the incidence angle *θ* for TM-polarized excitation. The measurement results are summarized in Fig. 3a, indicating the maximum THG yield at *θ*_*cr*_ ≈ 15° and *λ*_*R*_ = 1.44 µm, with the maximal signal amplitude enhanced by a factor of ~10^3^ as compared to the excitation of the same BIC under normal incidence (see the inset in Fig. 3a). Overall EF of the THG process reaches ~10^5^ with respect to the signal detected from a smooth Au film (Fig. 2d), while the corresponding thermal damage threshold of the nanobump array under its optimal angular excitation shifts down to ~20 MW cm^−2^ revealing elevated light localization and enhancement in the electromagnetic hot spots over the nanobump surface according to the calculated intensity profile (Fig. 2a). In order to make comparison in terms of THG efficiency, we should note that the theoretical calculation predict the enhancement factors of up to 10^7^ in graphene ribbons structures^35^. Nevertheless, the experimental data reported show lower values. The THG enhancement of 10^5^ compared to our work was observed in film-coupled plasmonic gratings^36^. Similarly, periodic one-dimensional plasmonic gratings supporting high-Q resonances have produced TH signal reaching 10^4^ enhancement compared to a planar film^37^. Another illustrative example is the use of discontinuous “island” silver nanoparticle films, which, as a result of localized plasmon resonances, have yielded THG intensities exceeding those from homogeneous silver films by a factor 10^2^ ^38^.

Figure 3b provides the calculated dependence of the retrieved radiative and non-radiative losses of the plasmonic BIC on the incidence angle *θ* corresponding to the lateral wavevector *k*_*|*|_ = *k*_0_sin(*θ*). Critical angle obtained via the nonlinear optical response of the nanobump array appears to be larger (*θ*_cr_ ≈ 15°) as compared to calculated value (*θ*_cr_ ≈ 8°; dashed curve in Fig. 3b). One of the main reasons for this discrepancy is the underrated non-radiative losses of the BIC that were initially assessed in the simulations considering only the intrinsic Ohmic losses of gold (defined by imaginary part of permittivity *ε″* ≈ 8.3^39^). The analysis of data indicates that the matching with the measured critical coupling angle requires approximately a threefold increase in the non-radiative losses. Two main mechanisms can contribute to the increased extrinsic non-radiative losses: (1) defects or imperfections of the fabrication process leading to random variations of the nanobump geometry and array pitch^40,41^, and (2) optical heating of the metasurface under the laser excitation. Imperfections at the fabrication stage can stimulate re-scattering of quasi-BIC into the free space modes, thus effectively increasing non-radiative losses by coupling to additional channels. The optical heating increases the temperature of the Au film, resulting in the enhanced electron-phonon scattering in the metal^42^ and thus increasing the imaginary part of permittivity responsible for the losses.

The contribution of both mentioned mechanisms can be considered by increasing the effective imaginary part of dielectric permittivity *ε″* according to the Drude-Lorentz model. Non-radiative losses of the BIC mode estimated using *ε″* ≈ 50 are plotted in Fig. 3b (solid green curve), providing perfect matching with the measured critical coupling conditions at *θ*_cr_ ≈ 15°. The same modified permittivity of gold was used to calculate the THG yield of the nanobump array as a function of the incident angle *θ* shown as a map on the Fig. 3b. The simulations were carried out in a two-step manner (non-depleting pump approximation^31^) where the nonlinear polarization *P*_*i*_(3*ω*) at the third harmonic was computed based on the field distribution at the fundamental wavelength (see “Materials and methods” section). The simulated THG yield showed a good match with the measured data in terms of the critical angle around 15°, the dispersion of the plasmonic BIC mode, as well as the overall EF of the THG signal.

Another very important effect is related to the temperature dependence of the third-order nonlinear susceptibility due to the hot electrons contributions^31,43,44^. As presented in the references, variation of *χ*^(3)^ coefficient can reach several orders of magnitudes. Nevertheless, we do not observe any evidence of thermal variation of nonlinear susceptibility, since in our simulations it was assumed to be a constant. It cannot provide the explanation of the critical angle shift shown in Fig. 3, but eventually could shed light on the origin of slight deviations between the modeled and experimentally measured SHG intensities. More detailed studies of the temperature dependence of nonlinear susceptibility in nanobump arrays are still to be carried out.

### HgTe QDs-based SWIR photodetector empowered by plasmonic BICs of the laser-printed metasurface

**Fig. 4:**
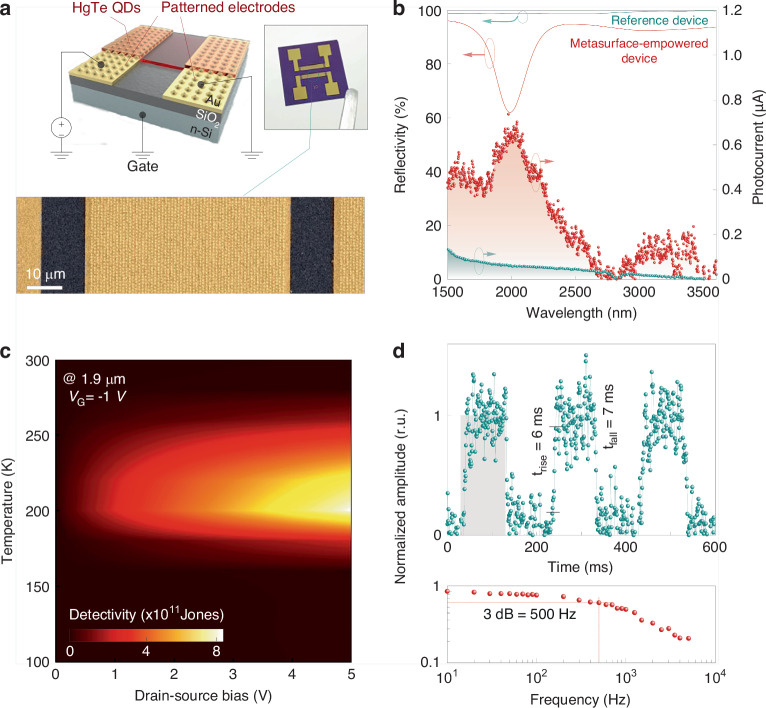
HgTe QDs-based photodetector empowered by laser-printed plasmonic BIC metasurface. **a** A 3D sketch, optical and false-color SEM image of the plasmonic nanobump array laser-printed on the drain and source contacts of the FET. **b** Reflection (solid curves) and photocurrent spectra (markers) of the HgTe QD photodetector with smooth (green) and patterned (red) Au electrodes. **c** Specific detectivity map (at 1.9 µm pump) as a function of the drain-source bias voltage and operating temperature. **d** Normalized current as a function of time (top panel) and modulation frequency (bottom panel) under excitation of 0.98 µm laser with 100 µs pulse width. Trigger electrical pulse is shown in gray color

Diverse metasurface architectures have recently been suggested as a promising solution to boost the performance of photodetectors using layers of semiconductor QDs as an active medium^45–52^. At the same time, there is still a high demand for further device improvements in terms of dynamic characteristics, uncooled operation, as well as inexpensive fabrication routes. We took advantage of the simplicity and accuracy of the laser fabrication process to directly produce the BIC plasmonic metasurfaces with *λ*_*R*_ = 1.95 µm and 2.5 µm over the Au electrodes of the off-the-shelf field-effect transistor (FET, see “Materials and methods” and Supplementary Materials). Schematics as well as optical and SEM images of the device concept are provided in Fig. 4a, where HgTe QDs with the absorption edge at 3 µm (Fig. S5) were directly spin-coated onto the modified and pre-cleaned FETs, forming a 50-nm-thick active layer. Note that the HgTe QDs were blended with In_2_O_3_ nanoparticles in a 10:1 volume ratio to optimize charge balance^53^ (Fig. S6). In its turn, the application of the QD layer slightly modified the effective refractive index of the BIC, resulting in a redshift of its spectral position up to 50 nm.

The effective coupling of HgTe QD absorption with the BIC of the plasmonic nanobump array (*λ*_*R*_ = 1.95 µm) resulted in a substantial enhanced absorption of the device active layer, achieving a 28-fold increase over bare QDs on smooth Au electrodes, as shown in Fig. 4b. Furthermore, the spectral photocurrent profile aligned well with the absorption maximum of the plasmonic BIC-empowered photodetector, ensuring a 13-fold enhancement near *λ*_*R*_ as compared to the reference unmodified device. Relatively lower enhancement of the photocurrent in relation to the increase in optical absorption (reflectivity drop) of the active QD layer can be caused by a significant dark current generated by holes due to the band alignment between slightly n-doped HgTe and Au electrodes^52^, as indicated in Fig. S6. Interestingly, one can observe a subtle increase in the photocurrent within 2.8 and 3.5 µm range, which can arise from the local plasmonic response of the nanobumps. In turn, combining the metasurfaces with the *λ*_*R*_ = 1.95 µm and 2.5 µm within one device resulted in a more comprehensive shaping of the photocurrent (Fig. S7), opening the possibility of simplified creation of broadband IR photodetectors.

Figures 4c and S7 provide responsivity and specific detectivity D^*^ of the BIC-empowered photodetectors in relation to operating temperature and applied bias voltage. At temperatures below 150 K, the responsivities of the plasmonic metasurface-integrated detector and the reference HgTe QD device were practically identical within the margin of error. By bias voltage and temperature regime tuning, it is possible to reach up to 240 and 10 A W^−^^1^ under 1900 nm and broadband blackbody (1250 K) illumination, respectively. Nevertheless, the rise of the dark current jeopardizes the responsivity value down to a few units toward room temperature. In turn, the trend in specific detectivity follows that of the responsivity, with the peak value of D^*^ calculated as (8.7 ± 1.2) × 10^11^ Jones at 5 V and 200 K for the series of four devices with *λ*_*R*_ = 1.95 µm. Meanwhile, under the same conditions, the champion broadband FET delivers specific detectivity to the 1250 K blackbody up to 2.5 × 10^11^ Jones. It is worth mentioning that in the range of 180 to 240 K, the detectivity shows two orders of magnitude enhancement for the plasmonic metasurface-integrated photodetector compared to the reference device (Fig. S7). Despite the superior values achieved, the specific detectivity is primarily influenced by the noise current density, which is significantly higher for the modified device compared to the reference one (Fig. S8). The 1/f noise is negligible for the reference HgTe QDs-based device at cryogenic temperatures (80 K), while the plasmonic metasurface-integrated photodetector exhibits a frequency-dependent impact up to 50 Hz. Nevertheless, it is not the dominant noise source, thanks to the intact surface of the ligand-exchange free HgTe QDs and the low bias voltage applied. Therefore, the Johnson (thermal) and generation-recombination noise should be taken into account. Given that the resistance of the photodetectors and insulating oxide quality were unaltered by the laser printing of the plasmonic metasurfaces, it could be deduced that thermal noise remains unaffected by the modification and could be mitigated by reducing the volume of HgTe QDs within the channel (Fig. S8). Conversely, higher concentration of generated charge carriers as well as scattering losses at the interface between the QDs and the electrodes modified with plasmonic metasurface might impact the generation-recombination noise of the devices^50–52^.

Finally, the transient response of photocurrent as well as the device operational bandwidth were measured (see Fig. 4d). A significant improvement in the detector’s response time was observed, attributed to the Purcell effect: specifically, the plasmonic BIC-empowered photodetector is characterized by rise and fall times of several microseconds while the reference device operates within the millisecond range. Although the response time improvement is not as extensive as previously reported for the plasmonics gratings-integrated photodiodes^54^, the values are comparable with other nanoantenna-like structures^50,52^. Meanwhile, the bandwidth of the modified detector is 500 Hz, hinting at the gain-bandwidth trade-off (Fig. 4d; bottom panel). The obtained results support the concept of benefiting from both enhanced optical absorption of the active layer and a faster response time, while maintaining a relatively simple and single-step approach to integrate BIC-supporting metasurfaces into QD-based IR photodetectors.

## Conclusion

We used a facile maskless and single-step procedure for the fabrication of the plasmonic metasurfaces through direct fs-laser modification of the substrate-supported Au films with the hollow protruding nanobumps. By probing the linear and nonlinear optical responses of the fabricated metasurface, we justified that the ordered arrays of the nanobumps support a collective resonant plasmonic response associated with a high-Q symmetry-protected BIC mode. Comprehensive optical modeling unveiled the field profile and angular dispersion of the BIC mode, suggesting the existence of the critical coupling conditions with matched radiative/non-radiative losses and enhanced absorbance/light-matter interaction. Being pumped under such critical coupling conditions, the metasurface demonstrated superior THG yield enhanced by a factor of 10^5^ as compared to the smooth Au film, proving the modeling results and justifying the efficiency of the BIC-supporting metasurface for light localization at the nanoscale and nonlinear conversion. We further utilized the developed fabrication route to create the HgTe QD-based photodetector with Au electrodes modified by imprinting plasmonic metasurface, and showed resonant enhancement of the photocurrent within the BIC spectral region as well as improved dynamic response. To the best of our knowledge, our work represents the first demonstration of the QD-based IR-photodetector empowered by the BIC-supporting plasmonic metasurface.

The demonstrated laser fabrication process provided high reproducibility thanks to the non-ablative nature of the laser patterning processes, while the spectral position of the BIC mode can be tuned within a broad near-to-mid IR spectral range (1.3–8 µm) by regulating the main laser patterning parameters and the array pitch. Considering the simplicity of the fabrication method, its scalability and tunability, the demonstrated BIC-supporting metasurfaces can find application in many advanced devices relying on resonant light-matter interaction, such as diverse photodetectors based on emerging materials and optical sensors, especially those based on the surface-enhanced IR absorption.

## Materials and methods

### Laser printing of plasmonic metasurface

Plasmonic metasurfaces were produced over a 50-nm-thick Au film surface using direct fs-laser printing. The film was coated over a monocrystalline Si wafer by e-beam deposition and textured by second-harmonic (515 nm) 200-fs laser pulses. Single laser pulse focused onto the film surface by a dry optical lens with a numerical aperture (NA) of 0.42 yielded a single protruding nanostructure. Fabrication of regular nanostructure array with a pre-defined pitch size was achieved by translating the laser beam along the sample surface using a PC-driven nanopositioning platform. Morphology of the produced nanostructured arrays was controlled through direct SEM visualization (Ultra 55+, Carl Zeiss) as well as by the preparation of the cross-sectional cuts using FIB milling (Thermo Fisher).

### Optical and nonlinear optical characterization

FTIR micro-spectroscopy setup (Vertex 70 v and Hyperion 2000, Bruker) was used to measure the near-IR reflectance of the laser-printed plasmonic metasurfaces. A home-built confocal optical microscope with a Fourier-plane imaging modality and coupled parametric oscillator (TOPOL, Avesta) generating near-IR fs-laser radiation (80 MHz, 1200–1600 nm tuning range) was used to pump plasmonic metasurfaces (see Fig. S9). The linearly polarized output pump beam was shaped by a set of lenses and focused by a dry lens (NA = 0.42) to irradiate the surface site with a diameter of about 50 µm. Nonlinear signal in the visible spectral range was collected from the exposed site by the same lens and analyzed by an optical spectrometer (Shamrock 303i, Andor) equipped with a TE-cooled CCD camera (Newton, Andor).

### Optical modeling

All numerical results were obtained using COMSOL Multiphysics 6.1 commercial software. The eigenmodes of isolated bumps and bump arrays were calculated based on the quasinormal mode (QNM) method^55^. For that, we employed an analytical Drude-Lorentz formula for the metal dispersion.

In order to calculate the absorptance map shown in Fig. 2b, an excitation port was positioned at the top of the computational domain, with the excitation wave configured to propagate along the *z*-axis while *x*, *y*-axes were aligned along the main axes of the nanobump array. The linear polarization of the excitation field was defined as transverse magnetic (TM) with **H** = (0, *H*_0_, 0), and transverse electric (TE) with **E** = (0, *E*_0_, 0). Here, *H*_0_ and *E*_0_ represent the incident amplitudes of the magnetic and electric components of the plane wave, respectively, and the angle of incidence is denoted by *θ*. Floquet periodicity conditions were applied at the computational domain boundaries.

To calculate the THG response shown in Fig. 3a, the computational process was divided into two distinct physical problems: (i) finding the field distribution at the fundamental frequency and (ii) the consequent solution of the problem at the frequency of the third harmonic. At the first step (i), the electromagnetic field distribution within the metasurface was obtained in the spectral range close to the BIC resonance wavelengths, while simultaneously varying the angle of incidence *θ*. The computational technique used at this step was the same as that employed for calculating the absorptance map shown in Fig. 2d. The electric field components at the fundamental frequency, *E*_*x*_(*ω*), *E*_*y*_(*ω*), and *E*_*z*_(*ω*), were used to build the nonlinear polarization vector of gold, which became the source of radiation at the second step (ii). The nonlinear polarization components were expressed as follows:

$$\left[\begin{array}{c}{P}_{x}(\omega )\\ {P}_{y}(\omega )\\ {P}_{z}(\omega )\end{array}\right]={\varepsilon }_{0}{\chi }^{(3)}\left[\begin{array}{c}{E}_{x}^{3}\left(\omega \right)+3{E}_{x}\left(\omega \right)({E}_{y}^{2}\left(\omega \right)+{E}_{z}^{2}\left(\omega \right))\\ {E}_{y}^{3}\left(\omega \right)+3{E}_{y}\left(\omega \right)({E}_{x}^{2}\left(\omega \right)+{E}_{z}^{2}\left(\omega \right))\\ {E}_{z}^{3}\left(\omega \right)+3{E}_{z}\left(\omega \right)({E}_{x}^{2}\left(\omega \right)+{E}_{y}^{2}\left(\omega \right))\end{array}\right]$$

Subsequently, at step (ii), the distribution of the electromagnetic wave at the third-harmonic wavelength was calculated. The total intensity of the THG was determined by integrating the Poynting vector over the outer surface of the computational domain.

### Fabrication of HgTe QDs-based FET photodetectors with laser-printed metasurface

#### Chemicals

Mercury chloride (HgCl_2_, Sigma-Aldrich, ACS Reagent), indium(III) acetate (In(Ac)_3_, Sigma-Aldrich, 99.99%), elemental tellurium (Te, Sigma-Aldrich, 99%), phosphoric acid (85%, diluted to 65% with deionized water, VWR Chemicals, ACS Reagent), 2-furanmethanethiol (FMT, Sigma-Aldrich, 98%), tripropylamine (TPA, Sigma-Aldrich, 98%), tetrachloroethylene (TCE, Aladdin, 99.5%), 1-dodecanethiol (DDT, AccuChem, AR grade), oleic acid (OA, Sigma-Aldrich, 90%), oleyl alcohol (Sigma-Aldrich, 85%), n-hexane (Anaqua, HPLC), formamide (FA, Unichem, GR grade), dimethylformamide (DMF, Duksan, GR grade), and high-purity Ar gas (99.999%) were all used as received.

#### HgTe QDs synthesis

HgTe QDs were produced following the procedure published previously^56,57^. The total volume of aprotic solvent (DMF) was 125 ml, divided between the bottom three-neck flask and the top funnel as 1:2 (v:v). In a three-neck flask, 4 mmol of HgCl_2_ and 12 mmol of FMT were dissolved in DMF. In a pressure-equalizing funnel, 3 ml of TPA was mixed with the rest of the solvent. Both precursor mixtures were deaerated with a flow of high-purity Ar gas bubbles for 1 h. H_2_Te gas was produced in an electrochemical cell (65% concentrated phosphoric acid as an electrolyte, platinum wire as anode, and fused elemental tellurium as cathode) at 300 mA current with 60% efficiency of the delivered tellurium precursor. The feeding time of the top solution with H_2_Te gas depended on the desired cation to anion ratio and was 100 min. The gas outlet from the funnel was vented into a measuring cylinder containing a 1 M NaOH solution in order to absorb any excess of H_2_Te gas not fully reacted. The temperature in the three-neck flask was set at −13 °C, and 15 ml of the telluride-rich solution from the funnel was dropped quickly into the mercury-containing flask. Then, the temperature was raised gradually to 85 °C along with the simultaneous controlled dripping of the rest of the tellurium precursor into the lower flask. The crude solution was subsequently cooled to room temperature using an ice bath. The obtained HgTe QDs were precipitated once with hexane and ethyl acetate (v:v = 1:2.5) and redissolved in the desired volume of DMF.

#### In_2_O_3_ nanoparticles synthesis

Indium oxide nanoparticles were prepared by the continuous slow injection synthesis previously reported by Cho et al.^58^. The nanoparticles were capped with oleic acid and colloid stable in non-polar solvent (hexane). To remove their original long-chain ligand, a previously published procedure was used^59^. A strong alkylating agent (e.g., Et_3_OBF_4_ or Me_3_OBF_4_) was added to the 2-phase mixture of In_2_O_3_ nanoparticles in hexane and DMF. This mixture was stirred overnight, and afterward, hexane was discarded. In_2_O_3_ nanoparticles in DMF were washed several times with toluene as an antisolvent.

#### Basic characterization of HgTe QDs

Transmission electron microscopy (TEM) was performed using a JEOL JEM-2100F microscope operating with an accelerating voltage of 200 keV; X-ray and ultraviolet photoemission spectra were measured on an Escalab Xi+ (Thermo Scientific) X-ray photoelectron spectrometer with an AlK_α_ source gun. The data collection area was 900 µm. PL spectra were recorded on an Edinburgh Instruments FLS920P spectrometer system, equipped with a liquid-nitrogen-cooled InSb photodiode, with an 880 nm CW solid-state laser as the excitation source.

#### FET photodetector fabrication

Commercially available FET substrates (Suzhou Jingxi Electronics and Technology Co.) were used as a basis for the photodetectors. The structure of the FET was the following: p-doped Si bottom gate electrode, 300 nm thick SiO_2_ layer, 100 nm thick pre-printed gold electrodes forming 13 conductive channels with the length of 10 µm and the width of 5 mm. Plasmonic metasurfaces were directly laser-printed on top of the gold drain and source electrodes. HgTe QDs or their blend with In_2_O_3_ nanoparticles in DMF were spin-coated on the cleaned FET substrates at two stage regime (1000 rpm for 20 s followed by 4000 rpm for 40 s), with spin-coating being repeated several times to reach the desired thickness. The devices were dried under primary vacuum for at least 2 h.

#### Electrical measurements

FETs were inserted into an Optistat DN2 cryostat (Oxford Instruments). Then the sample chamber was vacuumed and backfilled with dry argon (99.999% purity), repeating this process three times to ensure the space was free from oxygen and moisture. The measurements were conducted using two Keithley 2440 source meters controlled by a LabVIEW program to apply bias voltages to the gate-source and drain-source channels and record the current. The voltage scans were performed in the range from +5 to −5 V, each cycle starting and ending from 0 V. A Mercury iTC (Oxford Instruments Company) unit was used to control the cryostat temperature in the range between 300 and 80 K.

For photoresponse measurements, either a 1.9 µm thulium-doped CW fiber laser (Thorlabs) or a 1250 K blackbody source (Bentham) was used as the illumination source. The part of the blackbody spectrum shorter than 1.9 µm was cut-off with long-pass filter, while the optical absorption edge of HgTe QDs at 3 µm served as a longer wavelength limit. The responsivity, R (A/W), was calculated using the equation R = I_*pc*_/(P_*in*_ × A), where I_*pc*_ (A) is the photocurrent value, P_*in*_ (W cm^−2^) is the illumination intensity, and A is the active area of the device (cm^2^). Noise current density measurements were performed using an SRS570 low-noise current pre-amplifier and an SR830 Lock-in amplifier (Stanford Research Systems). The devices were biased from a switchable bank of batteries, and a LabVIEW interface program was used to collect the data.

The specific detectivity D^*^ (Jones) of the photodetectors was calculated using the formula ${D}^{* }={\rm{R}}\times \sqrt{A}/{S}_{n}$, where ${S}_{n}$ is the noise current spectral density (A/Hz^0.5^).

Spectrally resolved photocurrent was measured using a Bentham ISR-300 monochromator equipped with built-in current pre-amplifier. The Globar (1250 K, Bentham) was used as a light source. The incident beam was shaped by a combination of CaF_2_ lenses.

Transient photocurrent was measured under 1.9 µm illumination (thulium-doped CW fiber laser, Thorlabs) modulated by an optical chopper wheel (20 to 1000 Hz, Thorlabs) and using a 0.98 µm tunable pulsed laser diode (Newport) light source directly modulated with a pulse generator. A SRS570 low-noise current pre-amplifier (Stanford Research Systems) was used to amplify the signal further collected with Tektronix TBS C1000 digital oscilloscope.

## Supplementary information

Supplementary
