# Supplementary material for: Nonlinear light conversion and infrared photodetection with laser-printed plasmonic metasurfaces supporting bound states in the continuum
Source: Light Sci Appl. 2026 Jan 2;15:23. doi: 10.1038/s41377-025-02040-4 (PMC12757600; doi:10.1038/s41377-025-02040-4)
Supplement: Supplementary file 1 — Supplementary [file 41377_2025_2040_MOESM1_ESM.docx]

**Supplementary Information for Nonlinear Light Conversion and Infrared Photodetection with Laser-Printed Plasmonic Metasurfaces Supporting Bound States in the Continuum**

Dmitrii V. Pavlov^1^, Kseniia A. Sergeeva^2^, Albert A. Seredin^3^, Artem B. Cherepakhin^1^, Aleksandr A. Sergeev^4^, Anastasiia V. Sokolova^2^, Yuri N. Kulchin^1^, Alexey Yu. Zhizhchenko^1^, Mihail I. Petrov^3,a^, Aleksandr A. Kuchmizhak^1,5,b^, Andrey L. Rogach^2,c^

^1^Institute of Automation and Control Processes, Far Eastern Branch, Russian Academy of Sciences, 5 Radio Str., Vladivostok 690041, Russia

^2^Department of Materials Science and Engineering, and Centre for Functional Photonics (CFP), City University of Hong Kong, Kowloon, Hong Kong SAR, 999077 P. R. China

^3^School of Physics and Engineering, ITMO University, Saint-Petersburg, 197101 Russia

^4^Department of Physics, Hong Kong University of Science and Technology, Clear Water Bay Rd, Kowloon, Hong Kong SAR, 999077 P. R. China

^5^Pacific Quantum Center, Far Eastern Federal University, 8 Sukhanova str., Vladivostok 690041, Russia

^a^[m.petrov@metalab.ifmo.ru](mailto:m.petrov@metalab.ifmo.ru)

^b^[alex.iacp.dvo@mail.ru](mailto:alex.iacp.dvo@mail.ru)

^c^[andrey.rogach@cityu.edu.hk](mailto:andrey.rogach@cityu.edu.hk)

**Reproducibility of the BIC spectral position upon cyclic metasurface printing**

Figure S1. Representative series of the FTIR reflection spectra of the plasmonic nanobump arrays fabricated in a cyclic way under identical conditions. The series unveils the maximal deviation of the ∆*λ* position within 1420±34 nm and the resonance amplitude no larger than 5%.

To illustrate the reproducibility of the nanobump arrays, here we provide a series of spectra in Figure S1 obtained from different samples fabricated using the same parameters and conditions. We see that the results can be reproduced with high enough precision.

**Bic symmetry of electric field components**


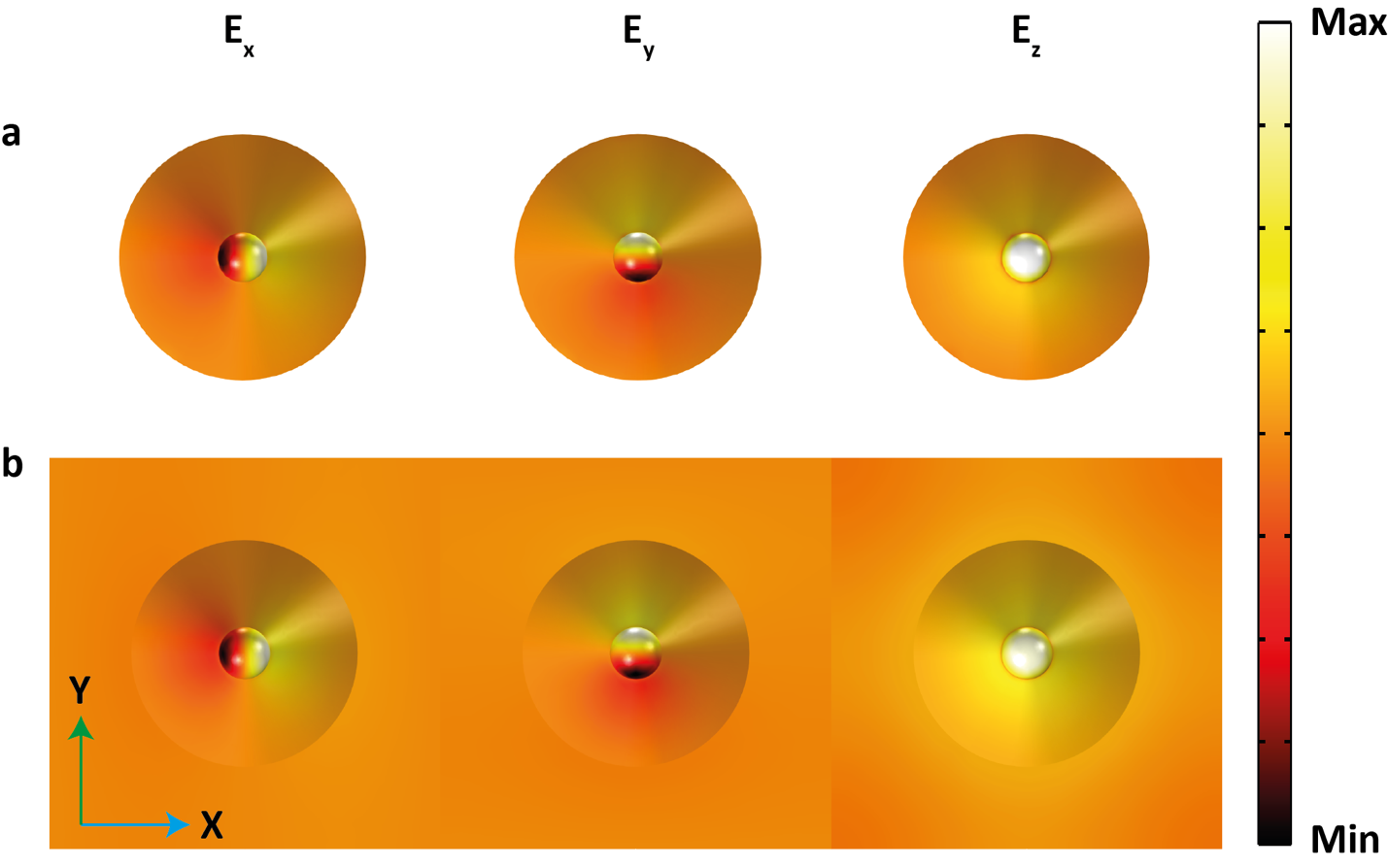


Figure S2. Distribution of electric field components of the isolated nanobump under excitation of its dipolar LSPR (a) and 2D array of such nanobumps under excitation of plasmonic BIC mode (b).

**The Effect of Surrounding Gold film in 2D array of nanobumps and plasmonic BIC behavior**

Periodic structures exhibit a linear dependence of the eigenmode wavelength on the period of the structure, as illustrated Figure S3a. Conversely, to gain a deeper understanding of how nanobumps exchange energy, one should examine the graphs in Figure S3b, which represent two different scenarios of nanobumps with a surrounding gold film (cyan line) and bumps without a gold film (magenta line), respectively. It is evident from these graphs that the quality factor of the plasmonic BIC mode for nanobumps with a gold film is significantly higher than for bumps without a surrounding film. This phenomenon can be attributed to the predominant transfer of energy between the nanobumps via surface plasmon polaritons. Furthermore, in Figure S3b (cyan line), one can observe an increase in the quality factor of the plasmonic BIC mode, accompanied by a decrease in the eigenfrequency. The rise in the quality factor is due to the increase in the propagation length of the plasmon polaritons as the wavelength lengthens. When the eigenwavelength of the plasmon mode shifts linearly with increasing array period (Figure S3a), the propagation length of the plasmon increases as a power function of the wavelength. This extended length propagation facilitates more efficient energy transfer between nanobumps via surface plasmon polaritons, which in turn reduces non radiative losses. Since the radiative losses are zero (all graphs are plotted for the eigenmode at the Γ-point), the total quality factor ultimately increases.


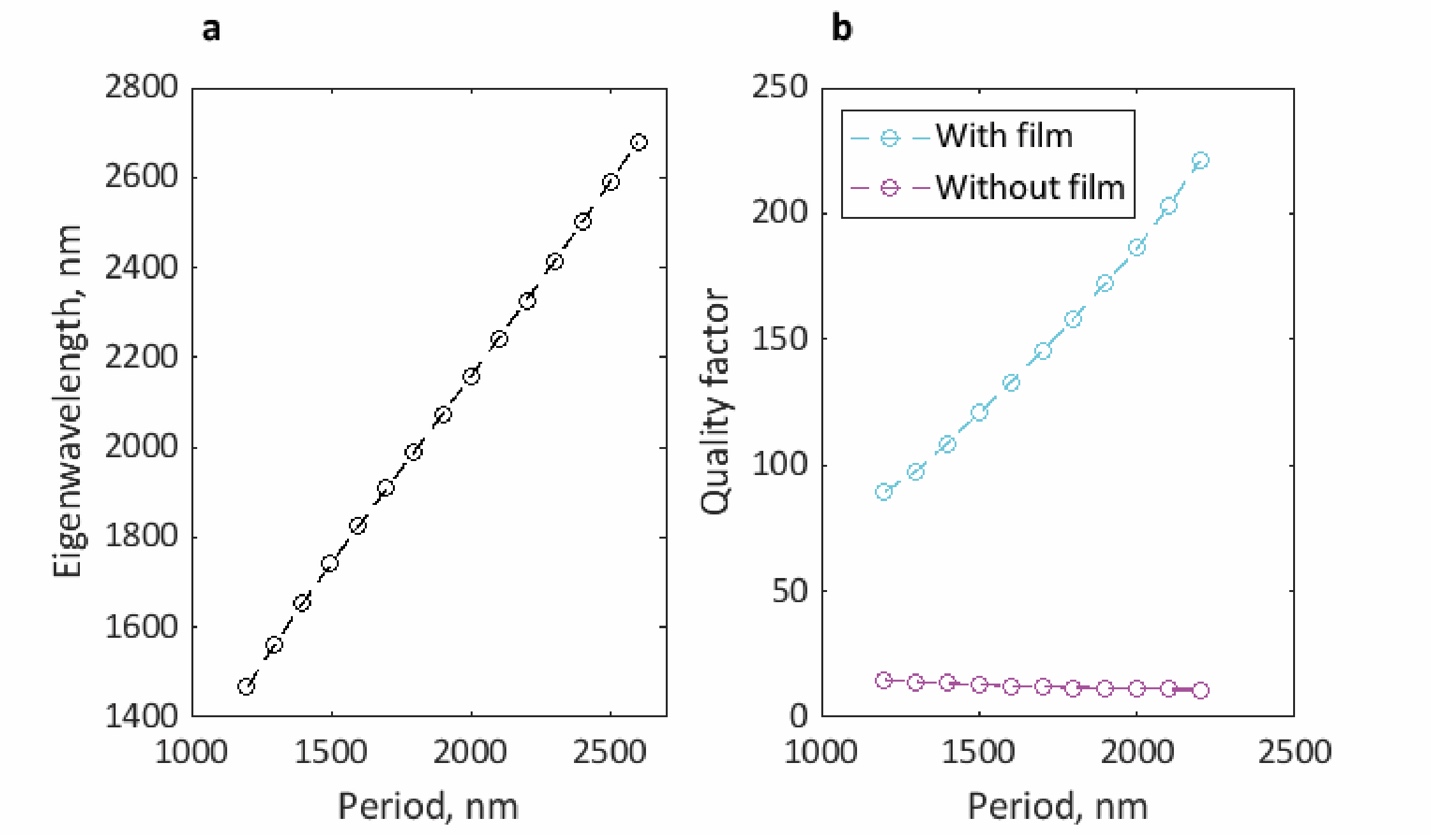


Figure S3. (a) Relationship between the eigenwavelength and the period of the metasurface for the plasmon BIC mode at the Γ-point. (b) Q-factor of the plasmonic BIC mode on the array period at the Γ-point for two different scenarios: the cyan line represents the case of the nanobumps connected by a gold film, while the magenta line denotes the case nanostructure array without the surrounding gold film.

**Dispersion Evolution**

In this study, we examine the interaction of a linearly polarized plane wave (TE, TM) with a plasmonic metasurface. This analysis is demonstrated through absorption maps as functions of the angle of incidence and wavelength for different bump sizes (relative heights H) (Figure S4). The first notable observation is the absolute insensitivity of the dispersion to the bump size for TE polarization in the metasurface regime (Λ/*λ* < 1), which can be attributed to the absence of heterogeneity in the magnetic permeability of the media. Secondly, by increasing the size of the bump, we enhance the amplitude of the periodic perturbation interaction, which leads to the enlargement of band gaps. Third, we observe a spectral broadening of the plasmonic BIC resonance (Λ/*λ* ≈ 0.8 corresponding to the mode discussed in the previous paragraph) at small angles of incidence (see Figure S4). It is important to note that the amplitude of the periodic disturbance determines the dominance of either local or non-local effects. Specifically, a large periodic amplitude results in the predominance of local effects, while a small periodic amplitude leads to the dominance of nonlocal effects.


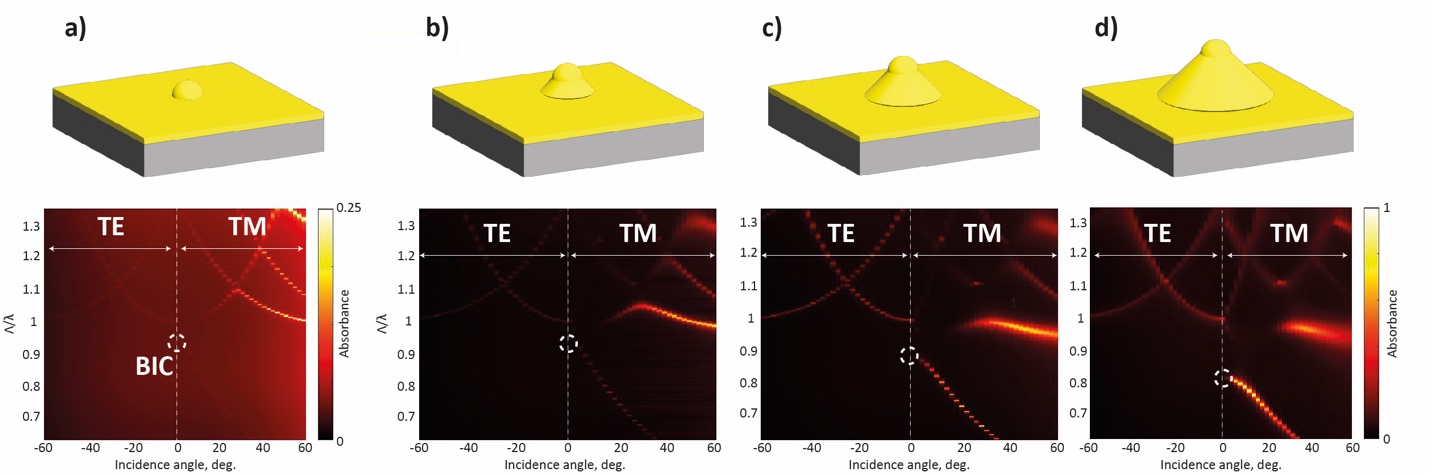


Figure S4. Absorption maps (left side of graph is for TE polarization, right side is for TM polarization) for various relative sizes of bumps: $\frac{1}{3}$H, $\frac{1}{2}$H, $\frac{2}{3}$H, and H (panels a, b, c, and d, respectively). The abscissa axis represents the angle of incidence of the plane wave with the corresponding polarization, while the ordinate axis denotes the ratio of the cell period to the wavelength (for b,c,d colorbar is common).

**IR photodetector characterization**


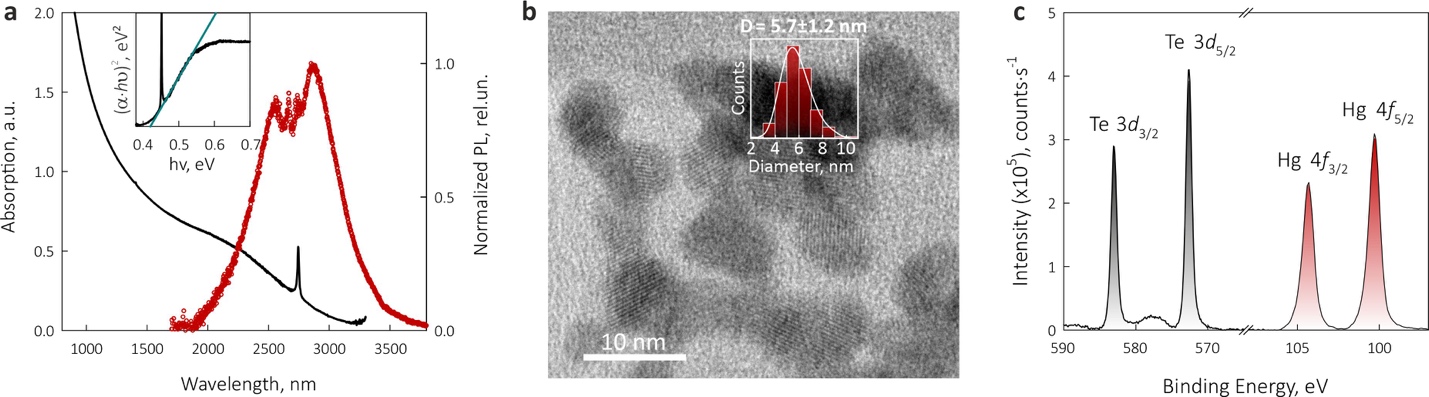


Figure S5. (a) Absorption and photoluminescence spectra of HgTe QDs. The inset shows the optical band edge defined using the Tauc plot. (b) TEM image of HgTe QDs and their size distribution (inset) calculated taking into account approximately 200 QDs. (c) XPS spectra of the Te-3d and Hg-4f core levels of HgTe QDs.


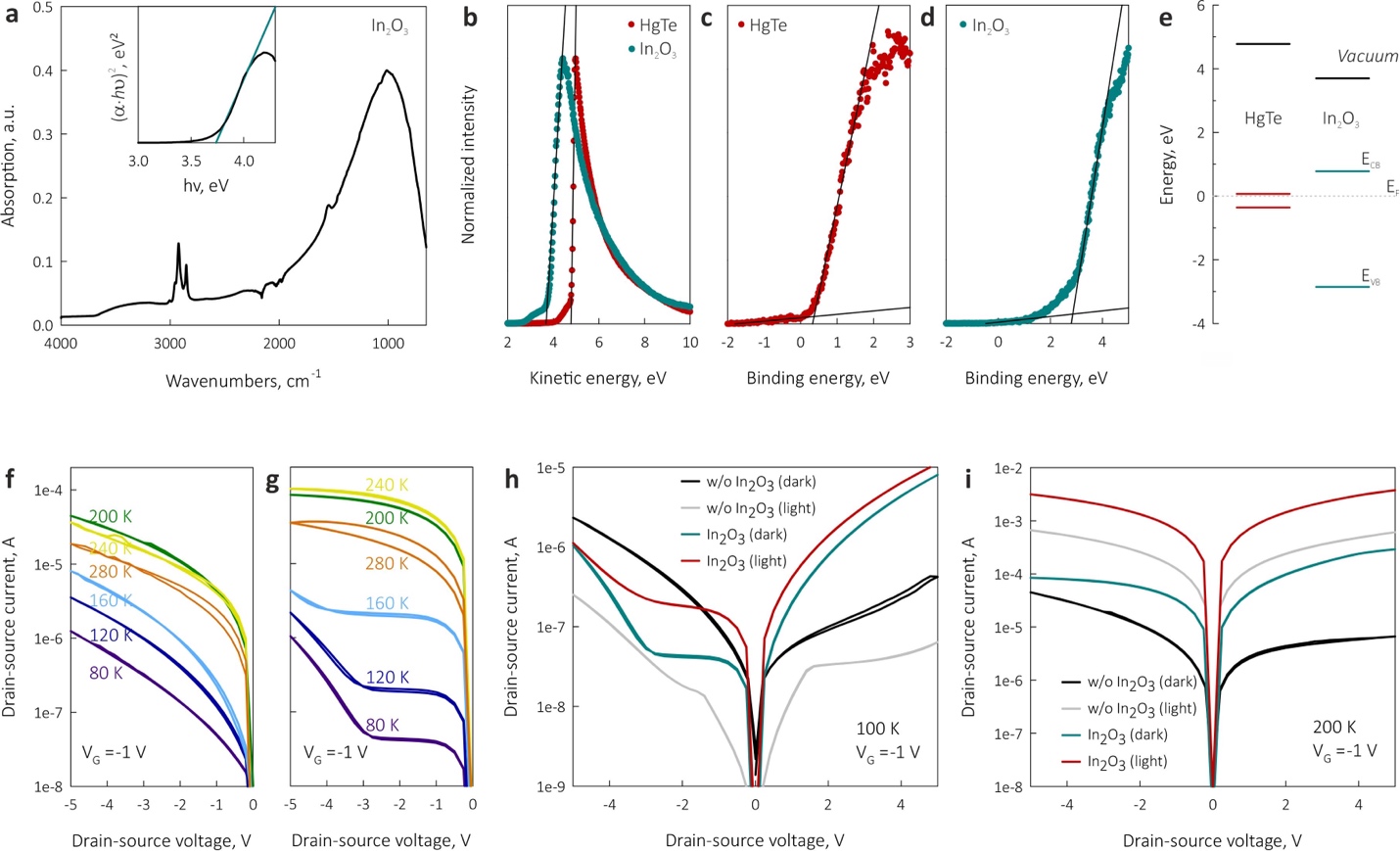


Figure S6. (a) IR absorption spectrum of the In_2_O_3_ nanoparticles. The inset shows the optical band edge defined using the Tauc plot. (b-e) UPS spectra of the HgTe QDs and In_2_O_3_ nanoparticles, and their mutual band alignment. (f) Dark current as a function of temperature of the HgTe QDs/plasmonic metasurface photodetector and (g) the photodetector fabricated by blending HgTe QDs with In_2_O_3_ nanoparticles. (h) Dark and under light (1.9 µm) currents of plasmonic nanobump-integrated devices with and without In_2_O_3_ nanoparticles at 100 K and (i) 200 K.


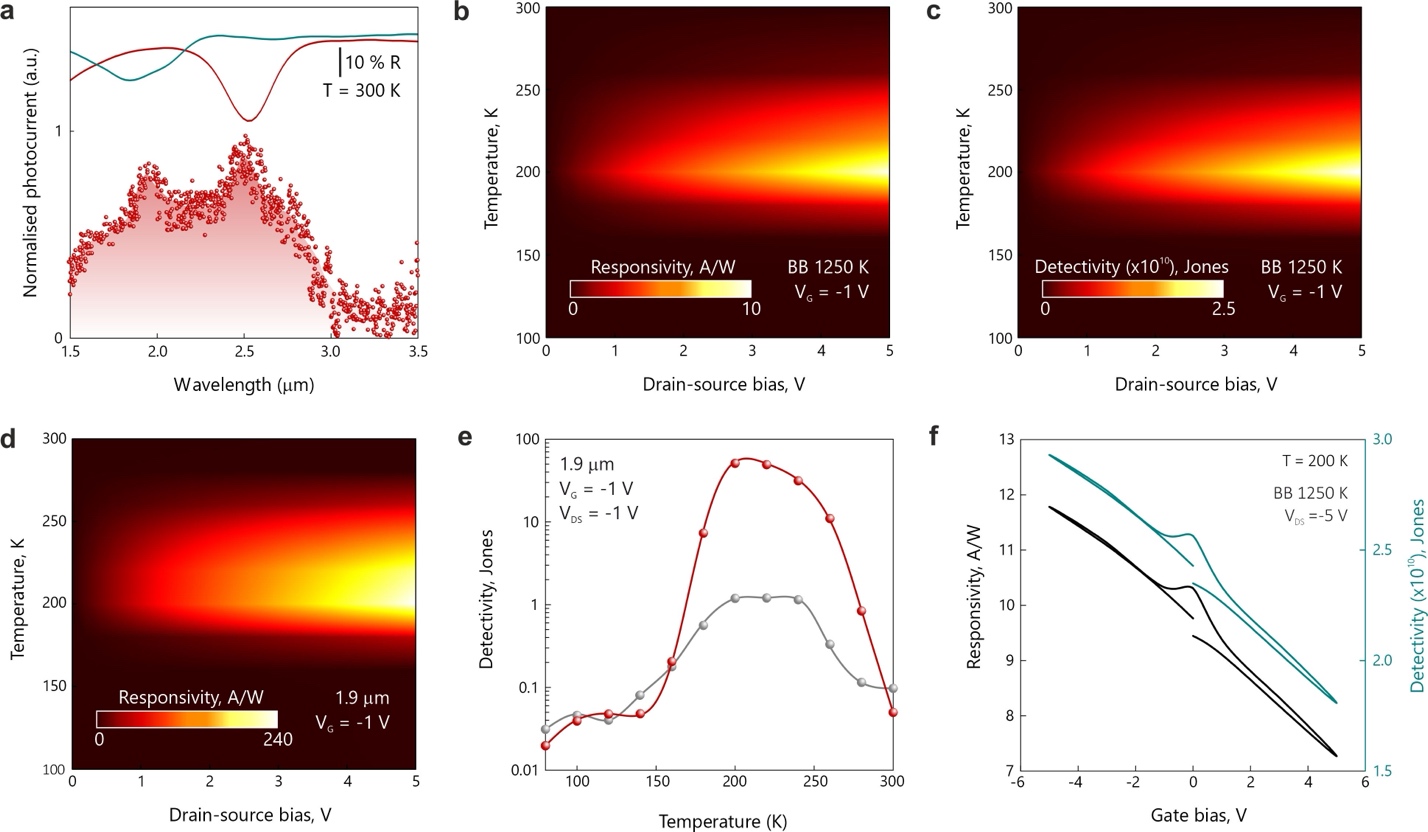


Figure S7. (a) Reflection spectra of the HgTe QD active layer on top of the nanobump arrays, and the corresponding photocurrent. (b-d) Responsivity and specific detectivity maps in relation to the operating temperature, bias voltage and illumination source for the HgTe QDs: In_2_O_3_/plasmonic metasurface photodetector. (e) Comparison of the performance of the reference (grey) and plasmonic metasurface-integrated (red) photodetectors. (f) Gate voltage tunability of the responsivity and detectivity for the HgTe QDs:In_2_O_3_/plasmonic metasurface photodetector operated at 200 K and V*_DS_* = 5 V.


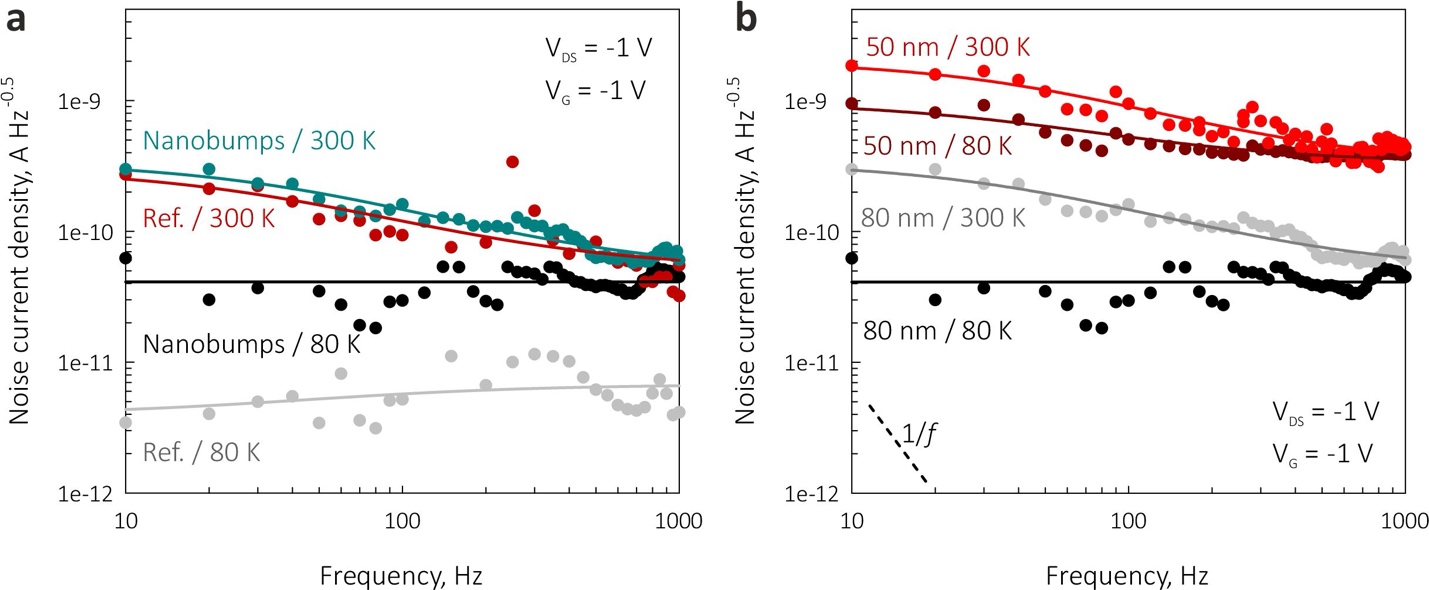


Figure S8. Noise current density of the plasmonic metasurface-integrated FETs (a) compared to the reference HgTe QDs device and (b) depending on the active QD layer thickness.

**Measurements of the THG yield of the BIC-supporting plasmonic metasurface**

Figure S9. Schematics of the setup used for THG experiments.
